# The safety and feasibility of laparoscopic right posterior sectionectomy vs. open approach: A systematic review and meta-analysis

**DOI:** 10.3389/fsurg.2022.1019117

**Published:** 2022-10-17

**Authors:** Meng-Xiao Wang, Ji-Feng Xiang, Sheng-Kai Chen, Lin-Kang Xiao

**Affiliations:** ^1^ Department of General Surgery, Chongqing General Hospital, Chongqing, China; ^2^ Department of Hepatopancreatobiliary Surgery, Institute of Hepatopancreatobiliary Surgery, Chongqing General Hospital, Chongqing, China

**Keywords:** hepatectomy, laparoscopic, hepatocellular carcinoma, laparoscopic right posterior sectionectomy, open right posterior sectionectomy

## Abstract

**Background:**

Laparoscopic right posterior sectionectomy (LRPS) is one of the most technically challenging and potentially hazardous procedures in laparoscopic liver resection. Although some available literature works demonstrated the safety and feasibility of LRPS, these data are limited to reports from a single institution and a small sample size without support from evidence-based medicine. So, we performed a meta-analysis to assess further the safety and feasibility of LRPS by comparing it with open right posterior sectionectomy (ORPS).

**Methods:**

MEDLINE, Embase, and Cochrane Library were systematically searched for eligible studies comparing LRPS and open approaches. Random and fixed-effects models were used to calculate outcome measures.

**Results:**

Four studies involving a total of 541 patients were identified for inclusion: 250 in the LRPS group and 291 in the ORPS group. The postoperative complication and margin were not statistically different between the two groups (OR: 0.49, 95% CI: 0.18 to 1.35, *P* = 0.17) (MD: 0.05, 95% CI: −0.47 to 0.57, *P* = 0.86), respectively. LRPS had a significantly longer operative time and shorter hospital stay (MD: 140.32, 95% CI: 16.73 to 263.91, *P* = 0.03) (MD: −1.64, 95% CI: −2.56 to −0.72, *P* = 0.0005) respectively.

**Conclusion:**

Data from currently available literature suggest that LRPS performed by an experienced surgeon is a safe and feasible procedure in selected patients and is associated with a reduction in the hospital stay.

## Introduction

Since the first total laparoscopic right posterior sectionectomy (LRPS) was reported in 2006 (1), it has motivated many surgeons for LRPS, and multiple case series have described its safety and feasibility (2, 3). In the era of innovating minimal invasive surgery, LRPS is a reasonable option to preserve remnant liver volume for lesions in the right posterior segment and has become an alternative to laparoscopic right hepatectomy (4). In addition, LRPS could increase the possibility of liver resection for recurrent hepatocellular carcinoma and reduce the risk of postoperative liver failure in cirrhosis (5). Like laparoscopic major hepatectomy, LRPS is considered a technically challenging procedure for its deeply anatomic location (6), scoring 9 or 10 on the difficulty scoring system of laparoscopic liver surgery (7). Owing to its technical demand and potential hazards, LRPS is only performed by experienced surgeons in large centers. There have been several published case series and cohort studies comparing the surgical outcomes of the laparoscopic approach with those of the open approach for right posterior sectionectomy (8–11). Although the available literature demonstrates the safety and feasibility of LRPS, these data are limited to reports from a single institution and a small sample size without support from evidence-based medicine. In response, we performed a meta-analysis to further assess the safety and feasibility of LPRS by comparing it with open right posterior sectionectomy (ORPS).

## Methods

This systematic review was conducted by following the PRISMA guidelines, and the protocol was registered at PROSPERO with registration number CRD42021227817.

### Inclusion criteria

Studies eligible for further analysis had to meet the following criteria: (1) comparison of postoperative outcomes of the laparoscopic approach with those of the open approach for right posterior sectionectomy in patients with Segment 6 or 7 benign and malignant lesions; (2) objective evaluation of the operative time, hospital stay, and postoperative complications; and (3) studies published as full-text articles in English literature.

### Exclusion criteria

Studies were excluded based on the following: (1) lack of comparative data; (2) hand-assisted technique in the description of the surgical method; and (3) studies published as reviews, notes, letters, and case reports.

### Search strategy and data extraction

A systematic search was independently performed by two authors in the MEDLINE, Embase, and Cochrane Library electronic databases. We searched for studies published between January 2000 and January 2022 using the Medical Subject Heading (MeSH) terms “laparoscopic right posterior sectionectomy,” “open right posterior sectionectomy,” “laparoscopic liver resection,” “laparoscopic hepatectomy,” “laparoscopic segmentectomy,” and “hepatocellular carcinoma.” These terms were combined using Boolean operators “AND” and “OR.” The references of the retrieved articles were also hand-searched to check for additional studies.

Potentially relevant literature were assessed by two independent investigators to exclude those that did not match the inclusion criteria. The data extracted included details of the study design, the characteristics of study samples, and outcomes. Any disagreements on the data collected were resolved through consensus.

### Methodological quality

Nonrandomized studies were assessed by the Newcastle–Ottawa Scale (NOS), which included three factors: patient selection, comparability of the study group, and outcome assessment. A score of 0 – 9 was assigned to each study, and studies achieving a score of 6 or higher were considered high quality (12).

### Outcomes of interest

The primary outcome was the postoperative complication. Secondary outcomes included operative time, hospital stay, and margin.

### Statistical analysis

RevMan 5.3 software was used for statistical analysis. Dichotomous variables were expressed as odds ratio (OR) with a 95% confidence interval (CI) using the Mantel–Haenszel statistical method. Continuous variables were expressed as mean difference (MD) with a 95% CI using the inverse variance statistical method. Heterogeneity was measured using Cochran's *Q* tests and expressed as *I*^2^ statistics graded according to the Cochrane classification. The fixed-effects method was used in the presence of low or moderate statistical inconsistency (*I*^2^ ≤ 50%), and the random-effects method was used in high statistical inconsistency (*I*^2^* *> 50%). The mean and standard deviation were calculated from the median and interquartile range using a method described by Hozo et al. (13). *P* ≤ 0.05 was considered a statistically significant difference.

## Results

A total of 62 records were identified from the database search. After excluding duplicates and irrelevant articles, 12 studies were considered for full-text review. Of these, 4 studies involving 541 patients were finally included for the analysis. A flowchart of the analyzed studies was presented in Table 1. None of the included studies was a randomized controlled trial. The nonrandomized studies that achieved a score of 7–8 assessed by the Newcastle–Ottawa Scale were considered high quality, and these characteristics are shown in Table 2.

**Table 1:** The flowchart of the analyzed studies.

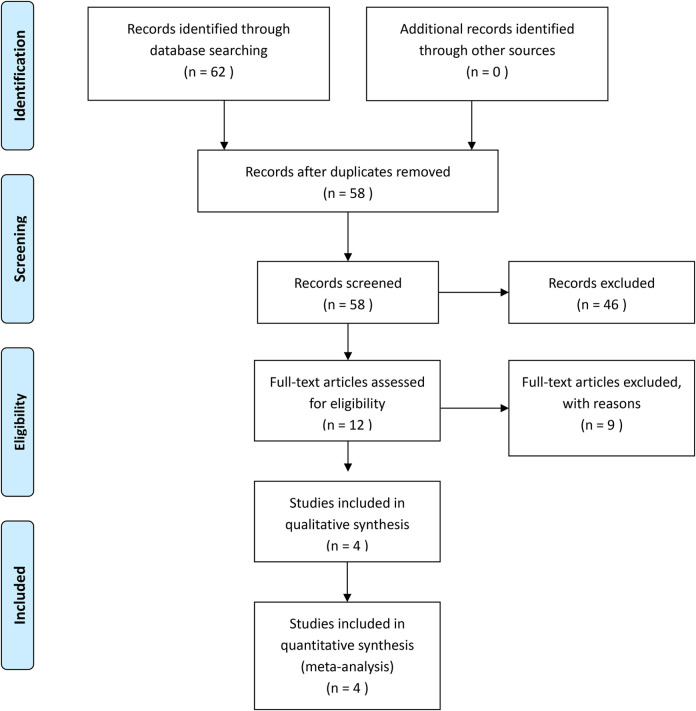

**Table 2:** Characteristics of included studies.

Study	Year	Country	Design	Patients		Age		Gender (M/F)		Tumor size	Quality score (0–9)
				LRPS	ORPS	LRPS	ORPS	LRPS	ORPS		
Dilai Luo	2020	China	Retrospective	23	25	56 ± 13	59 ± 11	15/8	18/7	3.2 ± 0.7	3.3 ± 0.6	7
Nicky van der Heijde	2020	Europe	Retrospective	150	150	64.5 ± 13.8	64.2 ± 13.5	87/63	85/65	49 ± 26	54/28	7
Jinsoo Rhu	2018	Korea	Retrospective	53	97	58.0 ± 8.8	58.2 ± 9.4	43/10	81/16	3.1 ± 1.8	3.1 ± 1.7	8
Jai Young Cho	2015	Korea	Retrospective	24	19	53.9 ± 12.6	60.0 ± 8.9	17/7	16/3	3.7 ± 1.8	4.8 ± 2.5	7

**Figure 1:**
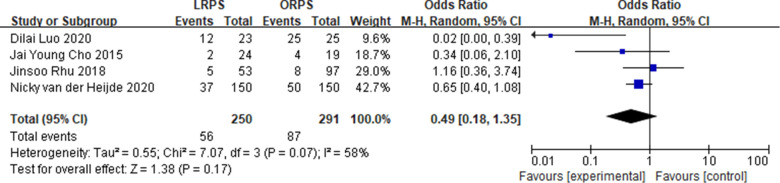
Pooled meta-analysis of postoperative complications comparing laparoscopic right posterior sectionectomy (LRPS) with open right posterior sectionectomy (ORPS).

**Figure 2:**
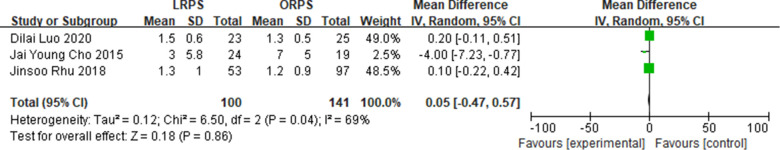
Pooled meta-analysis of margin comparing LRPS with ORPS.

**Figure 3:**
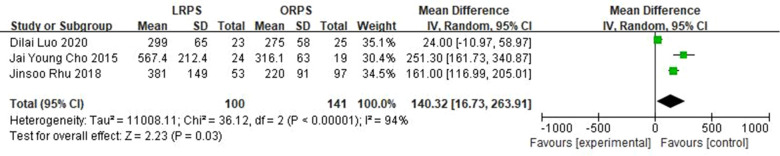
Pooled meta-analysis of operative time comparing LRPS with ORPS.

**Figure 4:**
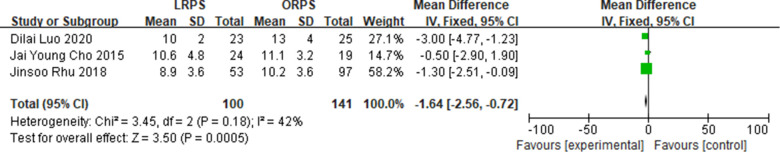
Pooled meta-analysis of hospital stay comparing LRPS with ORPS.

Four studies reported on postoperative complications, and three studies reported on operation time, hospital stay, and margin of the LRPS and ORPS procedures. The postoperative complication and margin were not statistically different between the two groups (OR: 0.49, 95% CI: 0.18 to 1.35, *P* = 0.17) (Figure 1) (MD: 0.05, 95% CI: −0.47 to 0.57, *P* = 0.86) (Figure 2), respectively. LRPS had a significantly longer operative time and shorter hospital stay (MD: 140.32, 95% CI: 16.73 to 263.91, *P* = 0.03) (Figure 3) (MD: −1.64, 95% CI: −2.56 to −0.72, *P* = 0.0005) (Figure 4), respectively.

## Discussion

With advances in devices and growing experience in the laparoscopic procedure, laparoscopic liver resection for lesions in the posterior segment has been achieved by some skilled surgeons (14, 15). Due to poor exposure to the surgical field and the difficulty of controlling hemorrhage during deep parenchymal transection, LRPS is classified as one of the most dangerous and challenging procedures and is even considered a contraindication in some centers (16). During the procedure of LRPS, the following difficulties and risks may arise: (1) the mobilization of the right lobe and transection of short hepatic veins around IVC has the risk of bleeding; (2) encircling the right posterior Glissonean pedicle is difficult and there is potential of bile duct injury; and (3) obtaining a correct cutting plane along the intersegment is also difficult. Despite this, some reports describe the safety and feasibility of the procedure and provide some innovative and effective surgical methods (17–19), and some even report laparoscopic anatomic resection of the right posterior sectionectomy (20, 21).

The main problems posed for LRPS are isolation of the right posterior Glissonean pedicle and the exposure of the right hepatic vein during parenchymal transection. The identification and isolation of the right posterior Glissonean pedicle are critical for LRPS, especially anatomic resection, and are indispensable to obtaining the ischemic demarcation that could facilitate maintaining a correct cutting plane along the intersegment. The right posterior Glissonean pedicle was concealed in the liver parenchyma, increasing the difficulty of dissection and the risk of injury to the right anterior Glissonean branch. Usually, the Rouviere sulcus approach was used to identify and isolate the right posterior Glissonean pedicle safely and easily (11, 16). In addition, there are other methods; for example, Machado et al. described a technique for LRPS using an intrahepatic Glissonean approach by small incision (5), and Homma et al. provided a novel method for LRPS using the caudate lobe-first approach to encircle the right posterior Glissonean pedicle safely and easily (17). Since the Glissonean branches of the right posterior section cross over the boundary between the right posterior section and the caudate lobe, they could theoretically be identified from the posterior by the caudate lobe-first approach and could be effectively encircled. The other technical difficulty for LRPS is the exposure of the right hepatic vein. The exposure of the right hepatic vein trunk was achieved by identifying the vein branch. When the branch of the right hepatic vein is exposed, one should not rush to ligate but gradually identify the trunk along the branch. In addition, the trunk of the right hepatic vein could be identified by dissecting the liver parenchyma at the right-most line of the inferior vena cava after ligation of the short hepatic vein and Makuuchi ligament. In addition, preoperative evaluation by 3D videography and intraoperative laparoscopic ultrasound could facilitate exposure of the right hepatic vein. During parenchymal transection, exposure to the surgical field was obtained by the gravity effect of the right posterior liver lobe and counter-traction of the right anterior lobe by the assistant. Maintaining the central venous pressure below 5 cmH_2_O was crucial to effectively reduce intraoperative bleeding for LRPS, which could be achieved by anesthesiological interventions or laparoscopic infrahepatic inferior vena cava clamping (22).

To overcome the shortcomings of conventional laparoscopy for lesions in the right posterior segment, the robotic system may be another advisable option. Because of the artificial instruments, the robotic system provides increased dexterity, a three-dimensional, magnified view of the operation, and decreases surgeon fatigue (23, 24), which may facilitate exposure to the surgical field and reduce the conversion rate. However, there are no comparative studies of this technique with an open counterpart with regard to right posterior sectionectomy.

In the present study, there was no significant difference in postoperative complications and mortality. LRPS had a significantly longer operative time and shorter hospital stay. The longer operative time of LRPS compared with that of OPRS may be related to the learning curve of the laparoscopic approach and the difficulty in hemostasis under laparoscopy, and the shorter hospital stay of LRPS may be due to minimally invasive and rapid recovery. The margin was not statistically different between the two groups after pooled analysis. This may indicate that LRPS could be performed for hepatic malignancies in the posterior segment. Due to the lack of sufficient original study data on blood loss, a pooled analysis could not be performed. The original study on blood loss showed that LRPS had significantly less blood loss than ORPS. This result may be related to the fact that laparoscopic visual field magnification and pneumoperitoneum pressure could reduce bleeding.

This study has several limitations. First, the number of patients included in this meta-analysis was relatively small, the heterogeneity of included studies was high, and no subgroup analysis was performed. Additionally, the study lacked the analysis of blood loss, long-term complications, and oncological outcomes. Finally, there were few included studies and no randomized controlled trial in the meta-analysis. Although the pooled analysis demonstrated that LRPS is a safe and feasible procedure and is associated with a reduction in hospital stay, the results may not be definitive. Hence, reliable results needed to be demonstrated by large-scale, multicenter randomized controlled trials.

In conclusion, data from currently available literature suggest that when LRPS is performed by an experienced surgeon it is a safe and feasible procedure in select patients and is associated with a reduction in hospital stay.

## Data Availability

The original contributions presented in the study are included in the article/Supplementary Material further inquiries can be directed to the corresponding author.
